# Bioremediation of toxic metals in mining site of Zamfara metropolis using resident bacteria (*Pantoea agglomerans*): A optimization approach

**DOI:** 10.1016/j.heliyon.2020.e04704

**Published:** 2020-08-14

**Authors:** Kalen Ephraim Audu, Shola Elijah Adeniji, John Solomon Obidah

**Affiliations:** a Biology Department, Ahmadu Bello University, Zaria, Nigeria; b Chemistry Department, Ahmadu Bello University, Zaria, Nigeria; c Microbiology Department, Modibbo Adama University of Technology, Yola, Nigeria

**Keywords:** Chemistry, Environmental science, Microbiology, *Pantoea agglomerans*, Bioremediation, Biosorption, Heavy metal

## Abstract

**Background:**

Various clean-up techniques for heavy metals have been suggested and practiced for its biosorption from the contaminated or pollutant soil by using chemical and physical methods. But most of the methods are hazardous to the environment and expensive. This study was on how to determine the potential of resident bacteria in the removal of heavy metals from contaminated soils in Abare situated in Anka Local Government of Zamfara State, Nigeria. Thus, this study employed bioremediation technique for removal of heavy metals.

**Results:**

The preparation of Culture media and Isolation of bacteria of the different contaminated soils were achieved by spread plate method. Whereas, concentrations of the heavy metals (Lead (Pb), Copper (Cu) and Iron (Fe)) were determined by Atomic absorption spectrophotometer (AAS. *Pantoea agglomerans* was used for biosorption experiment. The concentrations of Pb ranged between 1.328 ± 0.493 to 2.326 ± 2.093 mg/L, Cu 0.234 ± 0.117 to 1.054 ± 1.486 mg/L and Fe 18.498 ± 11.462 to 27.754 ± 57.510 mg/L. The optimum temperature for biosorption condition was found to be 35 °C. More so, the optimum pH of (7) was observed for maximum biosorption of Pb and Cu ions by *Pantoea agglomerans* which may be attributed to homeostatic phenomenon and the availability of metal binding sites on the biosorbents. Metal uptake biosorption percentage revealed that *Pantoea agglomerans* absorbed 99.6% of Pb, 60% of Cu and 96% of Fe.

**Conclusion:**

This study revealed that *Pantoea agglomerans* potential for bioremediation of the three metals.

## 1 Introduction

Heavy metals are conventionally defined as elements with metallic properties and an atomic number greater than twenty (20) and most common being Cd, Cr, Fe, Cu, Hg, Pb, and Zn [1]. Some of these metals are micronutrients essential for plant growth, such as Zn, Cu, Mn, Ni, and Co, while others have unidentified biological function, such as Cd, Pb, and Hg [2]. However, accumulation of heavy metals in soils is leading to higher risks due to leaching into ground and surface water, uptake by plants and direct or indirect intake by human population. When present at increased levels of bioavailability, both essential (Cu, Zn, Mn, Fe, Ni, Mo) and non-essential metals (e.g. Cd, Pb, Hg, Cr) are toxic [3]. Metals are present in the solid phase and in solution, as free ions, or adsorbed to soil colloidal particles [4]. Under certain environmental conditions, metals may accumulate up to toxic levels and cause ecological damage [5]. Metals like Hg, Pb, Cd and Cr are viewed as toxic, whereas, Cu, Ni, Fe, Co and Zn are not toxic at lower concentration but due to widespread use their level is increased in the environment which may lead to the serious concern on environment and global population [6]. Heavy metals affect the number, diversity, and activities of soil microorganisms. The toxicity of these metals on microorganisms depend on a number of factors such as soil temperature, pH, clay minerals, organic matter, inorganic anions and cations, and chemical forms of the metal. Heavy metals can damage cell membranes, alter enzymes specificity, disrupt cellular functions and damage the structure of the DNA. As such, high levels of heavy metal ions contamination have adversely affected microbial populations and their related functions [7]. Metal pollution also have harmful effect on biological systems and does not undergo biodegradation. Toxic heavy metals such as Pb, Co, Cd can be differentiated from other pollutants, since they cannot be biodegraded but can be accumulated in living organisms, thus causing various diseases and disorders even in relatively lower concentrations [8]. Heavy metals like Pb and Cd affect the endocrine system, causing alterations in physiological functions [9]. Heavy metals, with soil residence times of thousands of years, pose numerous health dangers to higher organisms. They are also known to have effect on plant growth, ground cover and have a negative impact on soil microflora [10].

However, due to global industrialization, war, and nuclear processes a large amounts of toxic compounds are frequently released into the biosphere [11]. The heavy metals released through the various industries as effluent, nuclear radiation and releases of heavy metals by other process in the environment may contaminate the soil [12]. Since soil is one of the most important environments for microbes which can be easily exposed to many pollutants, evaluating the effects of pollutants on the microbial population is very paramount [11].

Persistent heavy metal pollution poses a major threat to all life in Zamfara metropolis due to its toxic effects. Metal contamination particularly (Fe, Cu and Pb) have led to different types of medical problems like birth defects, cancer, skin lesions, growth retardation leading to disabilities, liver and kidney damage and a host of other maladies such as a contaminant, low solubility and due to the carcinogenic and mutagenic potential [13]. The problem mentioned above affect people residing near mining site in Abare, Anka Local Government Area, Zamfara State which is as a result of the illegal mining activities in the area. However various clean-up techniques have been suggested and practiced for the removal of heavy metals from the contaminated or pollutant soil by using chemical and physical methods. Removal of Cd and Hg from aqueous solutions, including phytoremediation, chemical precipitation, ion exchange, adsorption [9]. However, these methods are hazardous to the environment and expensive to employ.

One of the less expensive clean up technology is the bioremediation. In addition, it remediates the soil *in-situ* and avoids dramatic landscape disruption, and preserves the ecosystem. Therefore, this research aimed to study the potential of resident bacteria in the removal of heavy metals from contaminated soils.

## 2 Materials and methods

### 2.1 Study area

**Figure 1:**
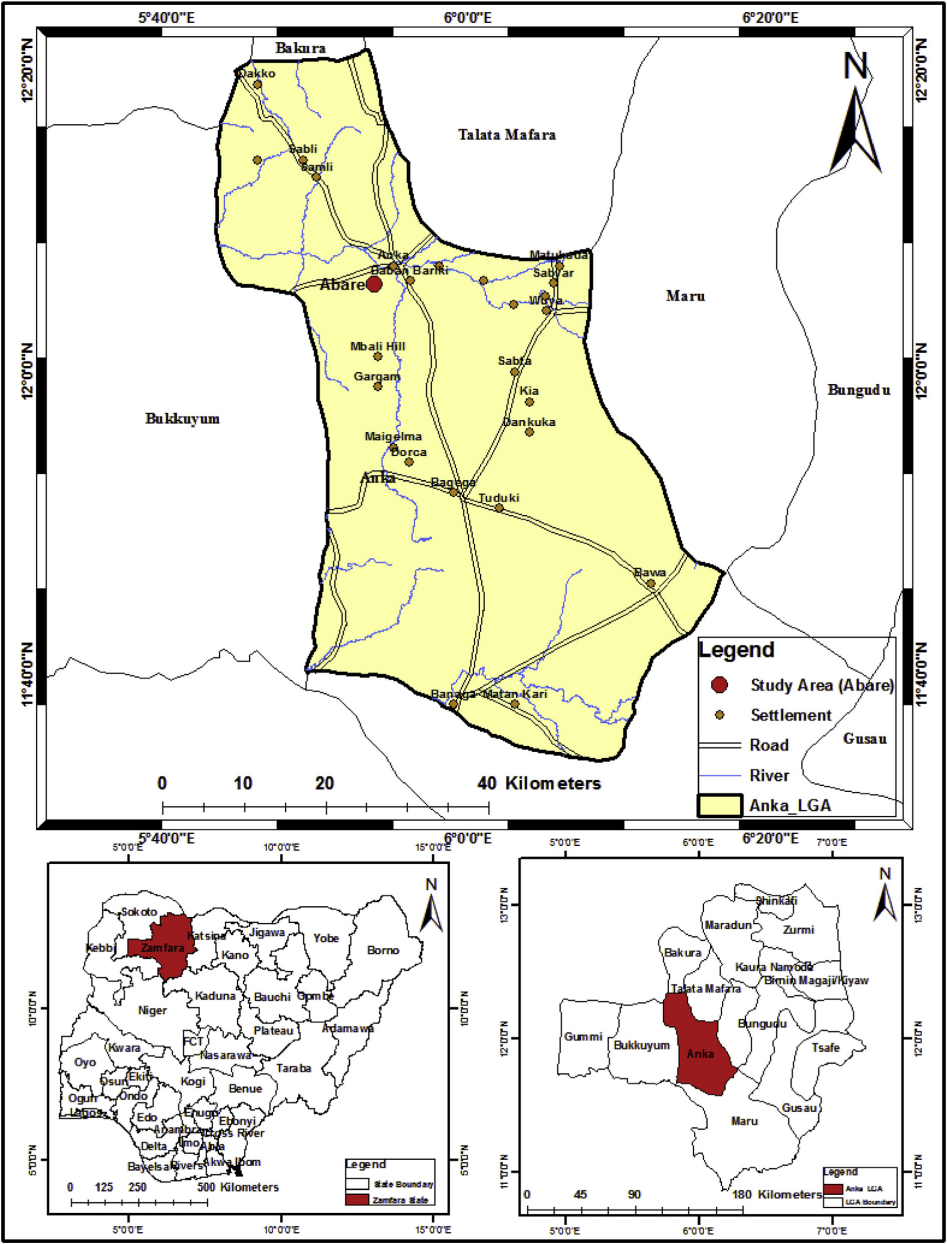
Map of the study area [15].

Abare is a remote village situated in Anka Local Governments of Zamfara State, Nigeria. It is a region where active artisanal gold mining is ongoing. It is located between latitudes 11º40′0″N and 12º0′0″ N and longitudes 6º00′E and 6º20′0'‘E of the equator. With an area of 2,940km2, Anka has a population of 263,400. The climate of Anka is warm tropical with temperatures rising up to 38 °C between March and May. Rainy season starts in late May to September while the dry season lasts from November to February. Two major soil types, ferruginous tropical soils and lithosols, dominate the local government [14]. The vegetation of the area consists of northern Guinea Savannah, characterized by short and stringy shrubs as seen Figure 1.

### 2.2 Sample collection and preparation

The soil samples were collected from three (3) different sites (Mining, Processing and Far from Processing Site). The samples were taking at a depth of 10–15 cm below soil surface using a soil auger. The samples were transfer onto labelled doubled clean polythene bags. The samples were air dry and sieved through 2mm sieve before laboratory analysis. 1g of the soil samples were dissolved in 10ml of water to make soil suspensions.

### 2.3 Preparation of culture media

Two different culture media were used in this study (Nutrient agar and Minimal medium). The nutrient agar medium was used for isolation of bacteria from soil sample. This medium contains the following; peptone, Sodium chloride and Beef extract. The final pH was adjusted at 7.0. Minimal medium was used to support the growth of isolates for bioremediation. This medium was containing; Yeast extract, CaCl_2_, K_2_HPO_2_, MgSO_4_, NaCl, KH_2_PO_4,_ and (NH_4_)_2_SO_4,_ at pH 7.0. All the above media were indented as grams per liter of distilled water, and subsequently autoclave sterilized at 121 °C for 20 min.

### 2.4 Preparation of stock solution of heavy metals

Different metal concentrations were prepared by dissolving Lead nitrate, Cupper penterhydrate and Ferrous sulphate salts in distilled water to get pure metal concentrations of Pb, Cu, and Fe. A stock solution of 1000 mg L-1 was prepared; all other concentrations (5ppm, 10ppm and 15ppm) were obtained from it.

### 2.5 Isolation of bacteria from contaminated soil

Bacteria was isolated from each soil sample and control sample. Nutrient agar medium was used for the isolation of bacteria from the soil sample. Serial dilutions from 10^−1^ to10^−5^ were prepared by pipetting 1ml of original suspension into 9ml normal saline. Then 1 ml of aliquot from 10^−3^ to10^−5^ dilutions were pipetted into sterile petri dishes containing 20 ml of nutrient agar. The plates were rotated slowly clockwise and anticlockwise at least 5 times to mix the suspension with nutrient agar. The plating was done in duplicate for each dilution. After setting the agar, the plates were incubated at 37 °C in an inverted position for 24 h. After which colonies with a clear zone of inhibition were observed.

### 2.6 Gram staining

Bacteria colonies from the plates were smeared aseptically on clean dried glass slides and gram stained following the standard procedure below. A drop of colony suspension was spread in the middle of a clean and grease free microscopic slide. Then the slide was air dried and passed through flame for fixation. Crystal violet was added for 1 min and washed with water. Gram's iodine was added for 1 min and washed with water. Then the preparation was decolorized with acetone for 30 s and washed with water. Safranin was applied for 1 min, washed with water and the slide was air dried. Using oil immersion, the stained slides were examined on the microscope under X100 objective lens.

### 2.7 Oxidase test

A piece of oxidase strip was divided into three equal section and labelled with the name of the isolates. A loop full of culture was rubbed on the moistened oxidase strip using sterile loop. The colour of the smear was checked exactly 15–30 s after rubbing the cells on the reagent moistened filter paper. A deep blue colour indicates positive reaction while a light violet or purple colour within 10 s indicates negative reaction.

### 2.8 Biochemical test using microgen ID system

All oxidase negative isolate was further confirmed using Microgen ID system as instructed by the manufacturer. Briefly, cell suspension was prepared by placing 1 colony in 3 ml of normal saline. The turbidity of the suspension was adjusted to 0.5 Mac Farland. The 0.5 Mac Farland was prepared by mixing 0.5ml of 1% Bacl_2_ and 9.95ml of 1% H_2_SO_4_ and agitated vigorously. The adhesive tape was removed and 3–4 drops (100μl) of the bacterial suspension were added to each well. Well 1 (Lysine), 2 (Ornithine), 3 (H_2_S) and 9 (Urease were overlaid with mineral oil. The top of the microwell test strip was sealed with the adhesive tape. Incubation was done at 37 °C for 18–24 h. After incubation the adhesive tape was removed and all positive reactions were recorded with the aid of the colour chart. Reagents were added to appropriate wells as follows: 2 drops of Kovac's reagent in Well 8 and read after 60 s, add 1 drop VPI and VPII reagent in Well 10 and read after 15–30minutes and add 1 drop of TDA reagent in Well 12 and read after 60 s. The final reading is recorded and analysized on Microgen software.

### 2.9 Determination of heavy metals content in the soil sample

Three heavy metals (Pb, Fe and Cu) in soil sample were determined. 1g of the soil sample was weighed and prepared for digestion. Digestion was carried out in triplicates of 5ml batches of the samples in a mixture of nitric acid and perchloric acid followed by heating at 100 °C for 45 min to 1 h to almost dryness and the volume made up to 60mlwith distilled water. The digest was filtered to remove insoluble material that could clod to the atomizer. The filtrates were then analyzed using Atomic Adsorption Spectrophotometry (Shimazu AAS, model AA-6800, Shimazu Corperation) at the National Research Institute for Chemical Technology (NARICT), Basawa, Zaria. Biosoption analyses were done at Department of Microbiology, Ahmadu Bello University, Zaria.

### 2.10 The effect of temperature

The isolates were inoculated into 3 set of designed 100 ml conical flasks containing different heavy metals (5 mg/L), minimal medium (50 ml) at pH 7 and inocula size of 6.0 × 10^8^ cfu/ml (Mcfarland turbidity standard).The flasks were incubated at different temperatures (30, 35, and 40 °C) for 24 h. After 24 h’ incubation, the biosorbent were separated by centrifugation at 3,000 rpm for 15 min and the remaining heavy metal concentrations determined using Atomic Adsorption Spectrophotometry (AAS). From the heavy metal removal efficiency, the optimum temperature was determined.

### 2.11 The effect of pH

The isolates were inoculated into 3 set of designed 100 ml conical flasks containing different heavy metals (5 mg/L), minimal medium (50 ml) and inocula size of 6.0 10^8^ cfu/ml (Mcfarland turbidity standard) at different pH (5, 7 and 9).The flasks were incubated at 35 °C for 24 h. After 24 h’ incubation, the biosorbent were separated by centrifugation at 3,000 rpm for 15 min and the remaining heavy metal concentrations were determined using Atomic Adsorption Spectrophotometry (AAS). From the heavy metal removal efficiency, the optimum pH was determined.

### 2.12 The effect of heavy metal concentration

The isolates were inoculated into 3 set of designed 100 ml conical flasks containing different heavy metals (5, 10 and 15 mg/L), minimal medium (50 ml), at pH 7 and inoculums size of 6.0 10^8^ cfu/ml (Mcfatland turbidity standard).The flasks were incubated at 35 °C for 24 h. After 24 h’ incubation, the biosorbent were separated by centrifugation at 3,000 rpm for 15 min and the remaining heavy metal concentrations were determined using Atomic Adsorption Spectrophotometry (AAS). From the heavy metal removal efficiency, the optimum concentration was determined.

### 2.13 Heavy metal uptake biosorption percentage under optimizes physiochemical conditions

The isolates were inoculated into a series of 100 ml conical flasks containing different heavy metals (5 mg L-1), 2g sterilize soil, minimal medium (50 ml) at pH 7 and inoculums size of 6.0 10^8^ cfu/ml (Mcfatland turbidity standard). The flasks were incubated at 35 °C) for 24 h. After 24 h’ incubation, the biosorbent were separated by centrifugation at 3,000 rpm for 15 min and the remaining heavy metal concentrations were determined using Atomic Adsorption Spectrophotometry (AAS). From the heavy metal removal efficiency, the optimum temperature was determined.

### 2.14 Statistical analysis

Statistical analysis of the metals concentration at different location was carried out by employing Analysis of Variance (ANOVA) with the aid of SPSS software (P≤0.05).

## 3 Results and discussion

### 3.1 Identification of bacteria isolated

The Isolate was selected based on their predominant growth on nutrient agar. Microgen GNA-ID system was used to confirm the species as *Pantoea agglomerans* with 84% probability and profile number 0164. This can be as a result of their cell wall being composed mainly by a thick layer of peptidoglycan which probably make them thrive well in metal contaminated environment.

### 3.2 Heavy metal content in the soil sample

The analysis of heavy mental concentration in the soil sample is reported in Table 1. It clearly shown that the average mean lead (Pb) level (1.32 mg/ml) in site A is less than (1.509 mg/ml) in the control site whereas, significant concentration of 2.936 mg/ml and 2.326 mg/ml in site B and C were found to be higher than the control site. For Copper Cu, site A (1.054 mg/ml) had the highest concentration compared to (1.509 mg/ml) in the control site whereas, concentration of 0.234 mg/ml and 0.264 mg/ml in site B and site C showed less concentration compared to the control site. For Iron, site C (38.382 mg/ml) and site A (37.754 mg/ml) showed higher concentration compared to 21.605 mg/ml in the control site. This results suggest that heavy meal toxicity in this contaminated soils has influenced the diversity of the bacterial community. The analyses of the heavy metals in the soils showed that there is a significant difference between the concentrations of metals found in all the three sites and the control site at p < 0.05. However, the high amount of Cu in site A can be attributed to the fact that the soil is intact with copper as an essential micronutrient required in the growth of both plants and animals, whereas the less amount of copper in site B could be associated with the processing activities which involved extracting gold along with Cu and Fe. In site B, lead (Pb) is high due to the fact that it is a waste product of mining and is being leached.

**Table 1:** Heavy meatal contents (mg) in the soil samples.

Location	Pb(mg)	Cu(mg)	Fe(mg)
Site A	1.328 ± 0.493	1.054 ± 1.486	37.754 ± 57.510
Site B	2.936 ± 1.851	0.234 ± 0.117	18.498 ± 11.462
Site C	2.326 ± 2.093	0.264 ± 0.127	38.382 ± 58.011
Control	1.509 ± 1.936	0.281 ± 0.220	21.605 ± 2.706

Confidence interval of 95%.

**Keys: Site A;** Mining Site, **Site B;** Processing Site, **Site C;** Far from Processing Site.

### 3.3 Optimization of the physiochemical conditions for biosorption

Biosorbents uptake potentials for heavy metals have been shown to depend on different physiochemical parameters. The results obtained for optimum temperature, pH and initial metal ion concentration for maximum removal of Pb, Cu and Fe ions by *P. agglomerans* were discussed as follows:

#### 3.3.1 Effect of temperature

**Figure 2:**
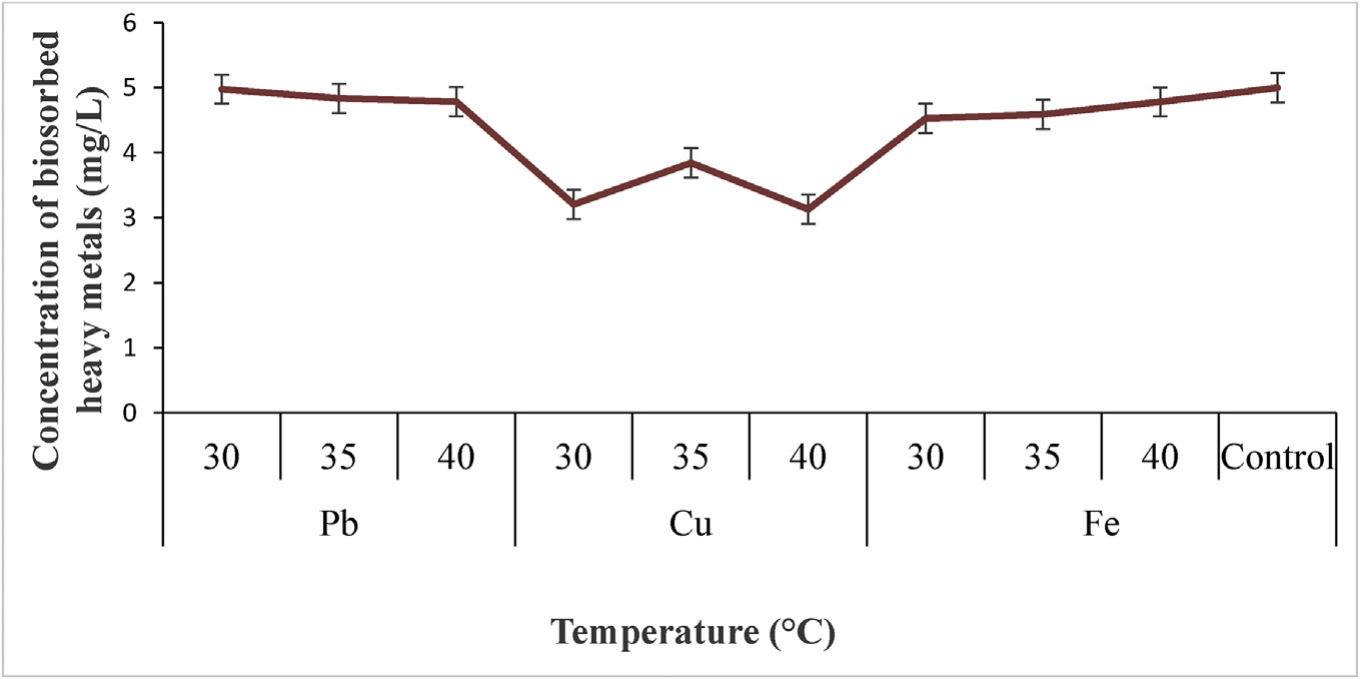
Biosorption potential of *P. agglomerans* for bioremediation of Pb, Cu and Fe at different temperature.

Optimum removal of heavy metal concentration (Pb, Cu and Fe) at of various temperature (30 °C, 35 °C and 40 °C) is presented in Figure 2. The optimum temperature for Pb ion removal by *P. agglomerans* was found to be 30 °C with maximum accumulation of 4.98 mg/l. However, it was observed that the optimum removal potential for Cu ions by *P. agglomerans* was 35 °C with maximum accumulation of 3.84 mg/l. Meanwhile optimum removal of Fe by *P. agglomerans* was observed at 40 °C with maximum accumulation of 4.78 mg/l. Comparatively, the result of this study showed no decrease in heavy metals concentration in the control at the varying temperatures. However, the trend of heavy metal removals by *P. agglomerans* at optimum temperature was,Pb > Fe > Cu (Figure 2). Key factors that affects microbial biosrption capacity for heavy metals is temperature. According to [16], temperature can affect the stability of microbial cell wall, its configuration and can also cause the ionization of chemical moieties. The variations observed in the present study, on the biosorption capacities of *Bacillus sp* for the heavy metals (Pb, Cu and Fe) at different temperatures was an evidence that heavy metals removal is temperature dependent [16]. reported an optimum temperature of 35 °C for effective biosorption of Cu ion by bacteria cell which is in agreement with the finding of this work for Cu by *Bacillus* sp. and for Pb ions biosorption by *Bacillus sp*. Moreover, the optimum temperature for maximum biosorption of Fe ions found in this work agreed with [17] who reported similar finding.

#### 3.3.2 Effect pH

**Figure 3:**
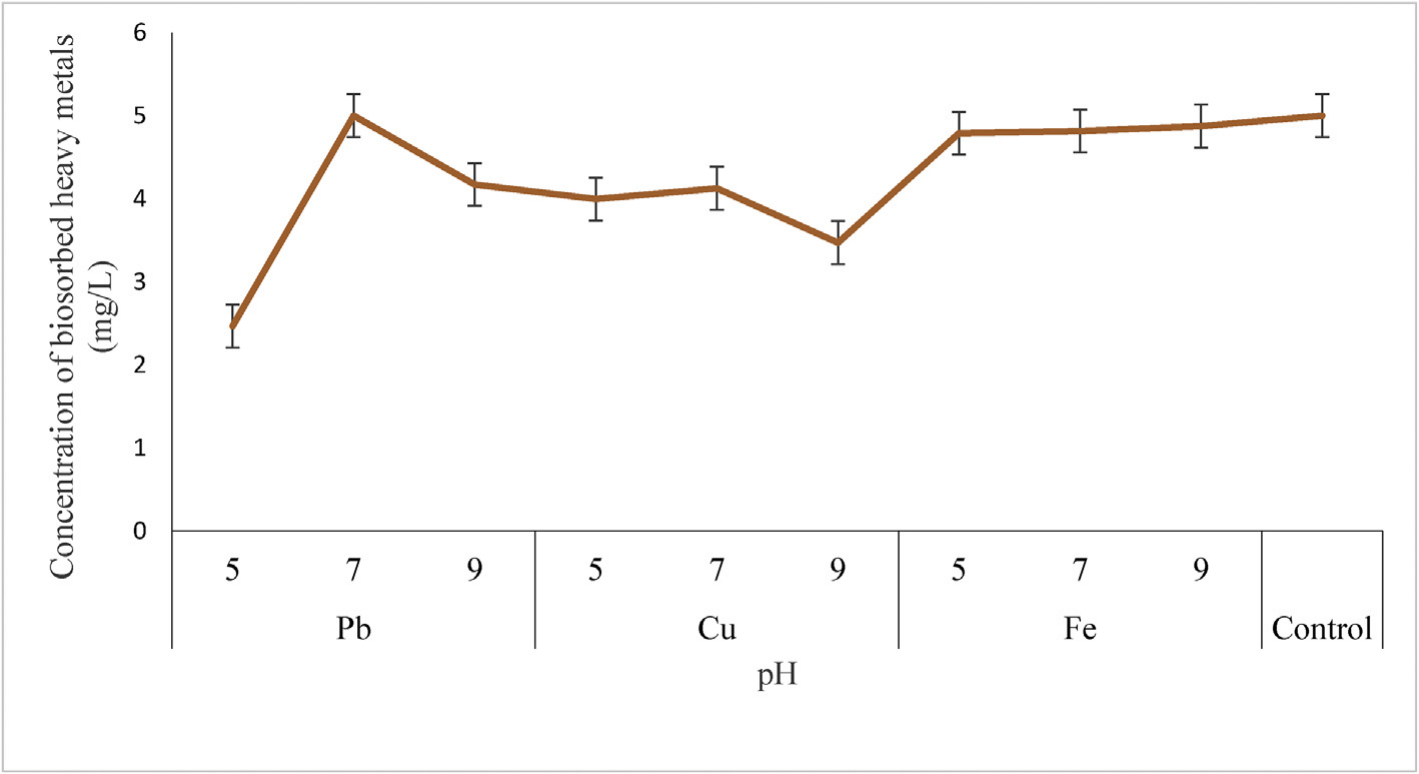
Biosorption potential of *P. agglomerans* for bioremediation of Pb, Cu and Fe at different pH.

The influence of pH on heavy metals biosorption capacity of *P. agglomerans* is presented in Figure 3. Result from the present study revealed pH of 7.0 as the optimum for Pb ion removal by *P. agglomerans* with highest accumulation of 5.000 mg/l. Moreover, pH of 7.0 was the optimum for Cu ion removal by *P. agglomerans* with highest accumulation of 4.124 mg/l. Meanwhile pH of 9.0 was recorded as the optimum for efficient removal of Fe ion by *P. agglomerans* with highest accumulation of 4.873 mg/l. However, the result of this study comparatively showed no decrease in heavy metals concentration in the control for all the varying Ph. In summary, the trend of heavy metals removal by *P. agglomerans* at optimum pH was,Pb > Fe > Cu [18]. opined that the affinity of cationic species for the functional groups present on the cellular surface of microbes is strongly dependent on the pH of the solution. This according to [19] is because, in the biosorption process, the pH affects two aspects: metal ion solubility and biosorbent total charge, since protons can be adsorbed or released. Our findings of different optimum pH for maximum biosorption of Pb, Cu and Fe ions by *P. agglomerans* indicates the pH dependency of the biosorbents and heavy metals for biosorption. More so, the optimum pH (7) recorded for maximum biosorption of Pb and Cu ions by *P. agglomerans* may be attributed to homeostatic phenomenon and the availability of metal binding sites on the biosorbents due to low protonation at this pH. This finding contradicted [20] who reported optimum pH of 3 and 5 for Pb and Cu biosorption respectively, using algal biosorbents. Reason may be due to differences in the types of functional groups on the biosorbents' cell surface. However, the optimum pH of 5 and 9 reported for iron maximum biosorption in this study may be as a result of decrease in competition between hydroxonium ions and metal species for the surface sites and also by the decrease in positive surface charge on the absorbents, which resulted in a lower electrostatic repulsion between the surface and the metal ion and hence uptake of metal increase at this pH. Our finding contradicted [21] who reported an optimum pH of 2 for maximum biosorption of Fe ion by algae. Possibly, this could also be for differences in types of functional group at the biosorbents’ cell surface.

#### 3.3.3 Effect initial concentration

**Figure 4:**
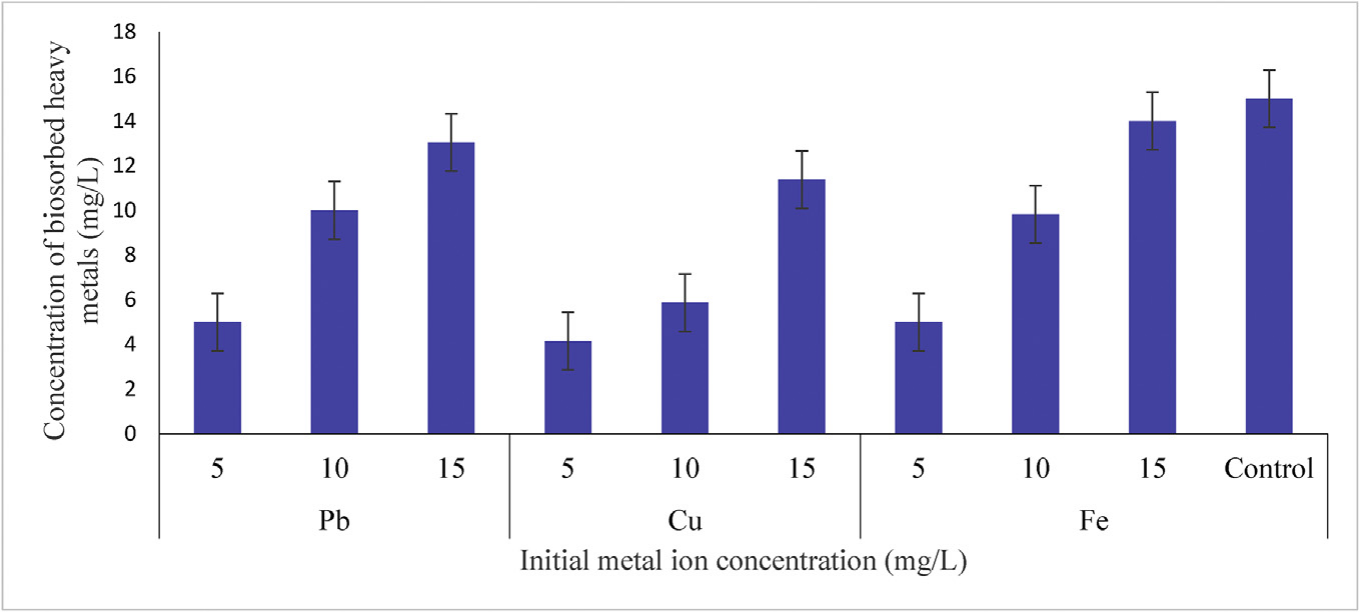
Biosorption potential of *P. agglomerans* for bioremediation of Pb, Cu and Fe at different Initial metal concentration.

Biosorption capacity of *P. agglomerans* at different initial concentrations of the heavy metals studied is presented in Figure 4. The result showed 15 mg/l as optimum initial concentration for Pb ion removal by *P. agglomerans* with maximum biosorption of 13.039 mg/l. Whereas, *P. agglomerans* removed Cu ion at optimum initial concentration of 15 mg/l with maximum biosorption of 11.369 mg/l. Furthermore, 14.000 mg/l was the most highly biosorbed concentration of Fe ion by *P. agglomerans* at optimum initial concentration of 15 mg/l. The trend of heavy metals removal at optimum initial concentration by *P. agglomerans* was, Pb > Fe > Cu. The initial concentration of metal ions provides an important driving force to overcome all mass transfer resistance of metal between the aqueous and solid phases [22]. Thus, in the present study, the increase in the amount of metal ions (Pb, Cu and Fe) adsorbed with increase in initial concentration at constant biomass (*P. agglomerans*) may be associated with the increase in the surface area of contact between the biosorbates and the biosorbents as initial metal ions concentration increases.

#### 3.3.4 Heavy metals uptake biosorption percentage under optimized condition in the soil

**Figure 5:**
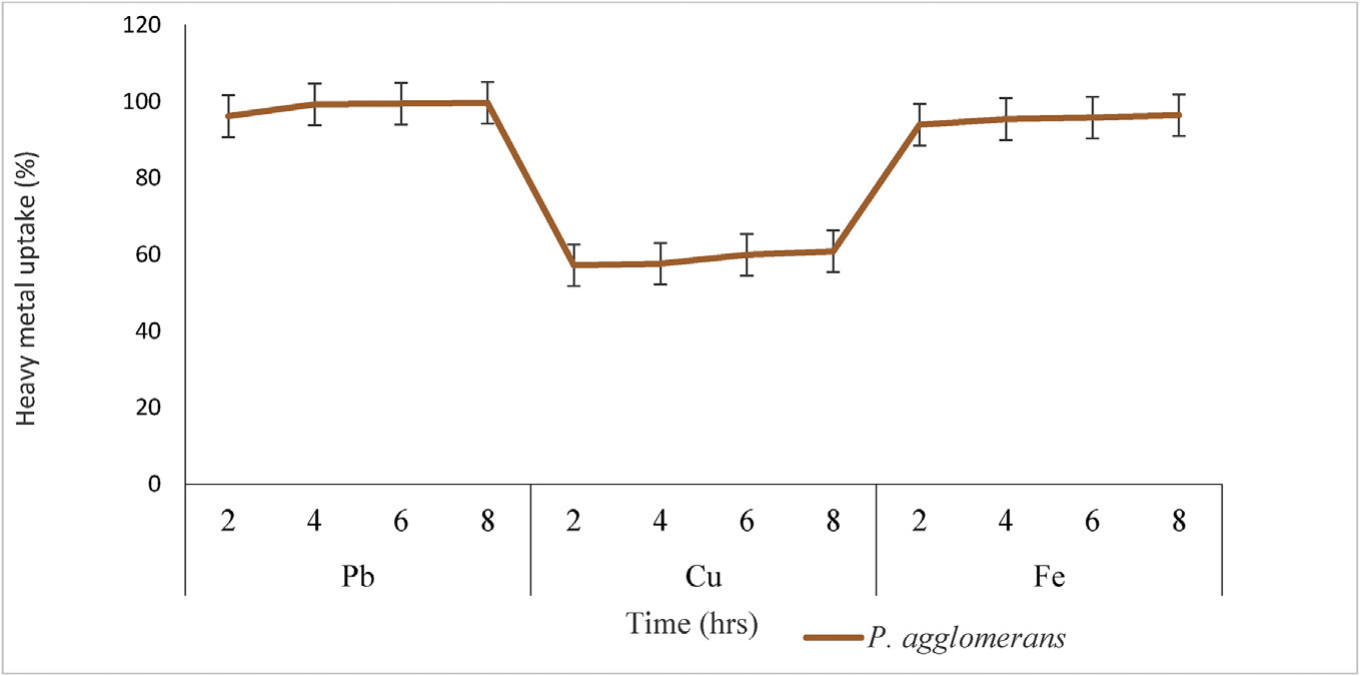
Heavy metal uptake biosorption percentage of *P agglomerans* at optimum conditions at different time.

Heavy metal uptake biosorption percentage of *P. agglomerans* is reported in Figure 5. The result of the study showed that, within 2hrs, *P. agglomerans* biosorbed 96.16% of Pb meanwhile, 99.24 % of Pb was removed by *P. agglomerans* within 4hrs. Moreso, within 6hrs, *P. agglomerans* removed 99.44% of Pb. *P. agglomerans* biosorbed 99.62% of Pb ion within 8hrs. The uptake of Cu ion however, revealed that, 57.18% was biosorbed by *P. agglomerans* within 2hrs, 57.058% of Cu ion within 4hrs. Within 6hrs, *P. agglomerans* took up 59.94% of Cu ion. Furthermore, *P. agglomerans* was able to removed 60.82 % of Cu ion within 8hrs. Results of Fe ion uptake showed that, *P. agglomerans* took up 93.92% within 2hrs. In addition, *P. agglomerans* removed 95.40% of Fe ion at approximately 4hrs. Moreover, within 6hrs, *P. agglomerans* took up 95.80% of Fe ion. In addition, *P. agglomerans* took up 96.44% of Fe ion within 8hrs. The variation observed in the uptake rate of a particular heavy metal by *P*. *agglomerans* could be attributed to the differences in the functional groups at the cell surfaces of the biosorbents. Also, the variation in the uptake rates of metal ions (Pb, Cu and Fe) by *P. agglomerans* could be linked to differences in the affinities of the metal ions to the functional groups at the cell surface of the biosorbent [23]. found similar variations when they studied the uptake rates of different metal ions by different biosorbents.

### 3.4 Molecular identification

In order to amplify the DNA of *P. agglomerans* on molecular level, universal primer was used to identify this strain. It appeared from our results that *P. agglomerans* was positive to 16S- 907r by PCR and detect 918 base pairs band on agarose gel electrophoresis as presented in Figure S1 in ESM. Because the 16S rRNA gene is common among all known bacteria, it serves as a primary reference for classification. In addition, the 16S rRNA gene is conservative and therefore allows design of universal primers [24]. The Primer 27f 907r corresponding to universal bacterial regions of the 16S rRNA gene of *P. agglomerans* were expected to amplify DNA from the chosen bacteria and the 16S rRNA parts were amplified. Amplicons for detection of universal bacteria (918 base pair long) was detected, indicating the presence of 16S rRNA gene. In this study, it was found that regions of high conservation spanned relatively high sequence variability of about 918 bp. The result is in agreement with the work of some researchers on the amplification of conserved sequence region in different bacteria by universal PCR technique for DNA sequencing or probe hybridization [24]. The work also agrees with the work of [23].

## 4 Conclusion

The present study isolated and identified soil bacterial composition in heavy metal contaminated soil. The strains representing morphologically different bacterial colonies were isolated and purified. The dominant gram negative populations belonged to the genus *Pantoea agglomerans* which accounted for more than 50 % of the total bacterial populations. The analyses of the heavy metals in the soils revealed the presence of Pb, Cu and Fe in varying concentrations.

Variations were observed in the biosorption capacities of *P. agglomerans* for the heavy metals (Pb, Cu and Fe) at different physicochemical conditions. *P. agglomerans* showed a biosorption trend of Pb > Fe > Cu at an optimum temperature of 35 °C. The biosorption trend observed at an optimum pH of 7 was Pb > Fe > Cu. At an optimum condition of 5 mg/L, *P. agglomerans* showed a biosorption trend of Fe > Pb > Cu. Bisorption percentage of *P. agglomerans* was studied at optimum conditions. The uptake of all the studied metals (Pb, Cu and Fe) were in the range of 2 < 4<6 < 8. Furthermore, *P. agglomerans* was observed to have the highest biosorption potential for Fe (96.44%).

This study shows that performing 16S rRNA PCR assays has the potential to make an important contribution by detecting the presence of bacterial gene in the isolates gene amplified.

## Declarations

### Author contribution statement

Kalen Ephraim Audu: Conceived and designed the experiments; Performed the experiments; Analyzed and interpreted the data; Contributed reagents, materials, analysis tools or data; Wrote the paper.

Shola Elijah Adeniji: Conceived and designed the experiments; Performed the experiments; Analyzed and interpreted the data; Contributed reagents, materials, analysis tools or data.

John Solomon Obidah: Conceived and designed the experiments; Performed the experiments; Analyzed and interpreted the data.

### Funding statement

This research did not receive any specific grant from funding agencies in the public, commercial, or not-for-profit sectors.

### Competing interest statement

The authors declare no conflict of interest.

### Additional information

No additional information is available for this paper.
