# Supplementary figures and images for: Bioremediation of toxic metals in mining site of Zamfara metropolis using resident bacteria (Pantoea agglomerans): A optimization approach
Source: Heliyon. 2020 Aug 14;6(8):e04704. doi: 10.1016/j.heliyon.2020.e04704 (PMC7452395; doi:10.1016/j.heliyon.2020.e04704)

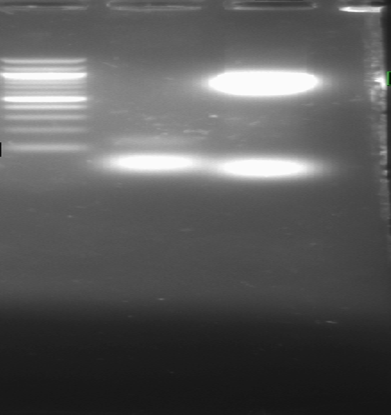


**918bp**

**Ladder**

**A2**

**100bp**

**1000bp**

Figure S1: Polymerase chain reaction product for P. *agglomerans* (A2)

Supplement: SUPPLEMENTARY_MATERIAL [file mmc1.docx]
